# Frequency of endometriotic lesions in peritoneum samples from asymptomatic fertile women and correlation with CA125 values

**DOI:** 10.1590/S1516-31802009000600004

**Published:** 2010-05-21

**Authors:** Caio Parente Barbosa, Ângela Mara Bentes de Souza, Bianca Bianco, Denise Christofolini, Fernanda Abani Mafra Bach, Geraldo Rodrigues de Lima

**Affiliations:** I MD, PhD. Regent professor, Division of Pathological Gynecology, Department of Gynecology and Obstetrics, Faculdade de Medicina do ABC, Santo André, São Paulo; and Gynecologist, Division of Gynecology, Department of Medicine, Universidade Federal de São Paulo (Unifesp), São Paulo, Brazil; II MD, PhD. Associate professor, Division of Pathological Gynecology, Department of Gynecology and Obstetrics, Faculdade de Medicina do ABC, Santo André, São Paulo, Brazil.; III PhD. Geneticist and collaborating professor, Division of Pathological Gynecology, Department of Gynecology and Obstetrics, Faculdade de Medicina do ABC, Santo André, São Paulo, Brazil.; IV Postgraduate student, Division of Pathological Gynecology, Department of Gynecology and Obstetrics, Faculdade de Medicina do ABC, Santo André, São Paulo, Brazil.; V MD, PhD. Titular professor, Division of Gynecology, Department of Medicine, Universidade Federal de São Paulo (Unifesp), São Paulo, Brazil.

**Keywords:** Endometriosis, Pelvic pain, Infertility, Dysmenorrhea, CA-125 antigen, Endometriose, Dor pélvica, Infertilidade, Dismenorréia, Antígeno Ca-125

## Abstract

**CONTEXT AND OBJECTIVE:**

Serological testing for CA125 has been widely used to detect endometriosis and to monitor its progression. However, controversy still exists regarding the usefulness of the plasma CA125 assay for diagnosing endometriosis. Furthermore, some authors have described superficial endometriosis as a cyclical and normal phenomenon in women’s lives, and have indicated that development and progression of this disease would only occur in some women as a result of immunological changes. This study aimed to determine the frequency of endometriosis and the correlation between serum CA125 levels and the presence of endometriotic lesions in the peritoneum of asymptomatic fertile patients.

**DESIGN AND SETTING:**

Cross-sectional study at the Family Planning outpatient clinic of Faculdade de Medicina do ABC.

**METHODS:**

Eighty asymptomatic fertile patients who underwent tubal sterilization surgery were studied. Blood and peritoneum samples were collected. CA125 levels were measured from blood samples, and peritoneum biopsies were studied using histopathological tests.

**RESULTS:**

Histopathological evaluation of the peritoneum revealed that 16.25% of the patients had minimal or mild endometriosis. There was no statistically significant difference in CA125 levels between patients with and without endometriosis.

**CONCLUSION:**

The presence of endometriotic lesions in the peritoneum of fertile patients supports the hypothesis that incidental findings of minimal or mild endometriosis may not be of clinical significance, and that the progression of the disease probably occurs as a result of immunological and genetic abnormalities. Serum CA125 levels did not show any diagnostic significance with regard to detecting the disease.

## RESUMO

**CONTEXTO E OBJETIVO:**

O teste sorológico para CA125 tem sido largamente usado para detectar e monitorar a progressão da endometriose, entretanto, a utilidade do ensaio do CA125 plasmático para o diagnóstico da endometriose ainda é controversa. Além disso, alguns autores descreveram a endometriose superficial como sendo um fenômeno cíclico e normal na vida de uma mulher, e que o desenvolvimento e progressão desta doença ocorreriam apenas em algumas mulheres como resultado de alterações imunológicas. Este estudo teve como objetivo determinar a freqüência de endometriose e a correlação entre os níveis séricos de CA125 e a presença de lesões endometrióticas em peritônio de pacientes férteis assintomáticas.

**TIPO DE ESTUDO E LOCAL:**

Estudo transversal realizado no Ambulatório de Planejamento Familiar da Faculdade de Medicina do ABC.

**MÉTODOS:**

Foram estudadas 80 pacientes férteis assintomáticas submetidas à cirurgia de esterilização tubária. Amostras de sangue e peritônio foram coletadas. Os níveis de CA125 foram medidos a partir das amostras de sangue e as biópsias de peritônio foram estudadas por ensaio histopatológico.

**RESULTADOS:**

O estudo histopatológico do peritônio revelou que 16,25% das pacientes apresentavam endometriose mínima e leve. Os níveis de CA125 não demonstraram diferença estatística significante entre pacientes com e sem endometriose.

**CONCLUSÃO:**

A presença de lesões endometrióticas em peritônio de pacientes férteis reforça a hipótese de que achados acidentais de endometriose mínima e leve podem não ter significância clínica, e que é provável que a progressão da doença ocorra como resultado de alterações genéticas e imunológicas. Os níveis séricos de CA125 não demonstraram significância diagnóstica para a detecção da doença.

## INTRODUCTION

An endometriotic lesion is defined as the presence of endometrial tissue outside the uterus, thus causing infertility, pelvic pain and dysmenorrhea.^1^ Estimates of the frequency of endometriosis range from 10-15% in women of reproductive age^2^ up to 50% in women with fertility problems^1^ and around 60%-70% in women with chronic pelvic pain.^3^ However, up to 20% of women may have endometriosis without presenting any symptoms.^1^

In clinical practice, severe endometriosis can be diagnosed with high accuracy by means of pelvic examination and through using imaging tools such as transvaginal ultrasonography^4^ and magnetic resonance.^5^ However, these methods are not sensitive enough to detect mild endometriosis,^6^ and laparoscopy or laparotomy is necessary in order to confirm the diagnosis and classify the stage of endometriosis according to the criteria of the American Fertility Society.^7^

Some authors have described superficial endometriosis as a cyclical and normal phenomenon in women’s lives, and have indicated that development and progression of this disease would only occur in some women as a result of immunological changes.^8^^,^^9^ Thus, the low specificity of the diagnostic methods available, along with the severity of the disease, has motivated new studies to search for noninvasive diagnostic methods to detection endometriosis.^6^

Serological testing for CA125 has been widely used to detect endometriosis and to monitor its progression.^10^ However, controversy still exists regarding the usefulness of the plasma CA125 assay for diagnosing endometriosis.^11^^,^^12^ Moreover, only a small and limited number of studies in the literature have combined peritoneal and serum samples,^3^ especially among asymptomatic fertile women.

## OBJECTIVE

This study aimed to determine the frequency of endometriosis and the correlation between serum CA125 levels and the presence of endometriotic lesions in the peritoneum of asymptomatic fertile patients.

## MATERIAL AND METHODS

### Patients

Eighty fertile women with no symptoms of endometriosis such as infertility, pelvic pain and/or dysmenorrhea (aged 21-44 years, mean: 33.68 ± 4.63) were studied. All of the subjects were attending the Family Planning outpatient clinic of Faculdade de Medicina do ABC (FMABC), Santo André, Brazil, and underwent surgery for tubal sterilization as a familiar planning measure.

The study protocol was approved by the local research ethics committee. All patients gave their informed consent for inclusion in the study.

## METHODS

### Histopathological evaluation

The patients underwent laparoscopic surgery for tubal sterilization and, during the procedure, small pieces of peritoneum were excised from four different sites: left and right ovarian fossae, and left and right sacrouterine ligaments.

**Figure 1.**
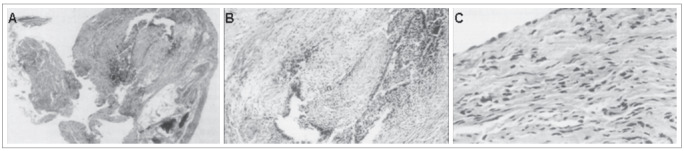
Photomicrographs at 100 X (A) and 400 X (B and C) of histological findings from peritoneum of asymptomatic fertile patients showing endometriosis (A and B), compared with normal tissue (C) (hematoxylin-eosin staining).

The biopsy specimens were fixed in a 10% formalin solution and embedded in paraffin. Histological sections (3-5 mm thick) were cut and stained with hematoxylin-eosin. A total of 320 microscope slides stained with hematoxylin-eosin were studied (Figure 1).

The criterion for histological classification of endometriosis was identification of stromal endometrioid or epithelial elements of Müllerian type, with or without stroma, associated with signs of hemorrhage and fibrosis.

After histological confirmation of the diagnosis of endometriosis, the lesions were classified morphologically, based on the variations in appearance of the ectopic endometrial structures.^13^ The morphological criteria for the analysis were: stromal disease, when only endometrial stroma was found; well-differentiated disease, when glands similar to topical endometrium were found; undifferentiated disease, when the appearance of the glands was different from topical endometrium; and mixed disease, when the appearance of the glands was atypical or undifferentiated.^14^ Endometriosis was staged according to the revised American Fertility Society (r-AFS) classification.^15^

### Serum CA125 level

Blood samples were collected on the first three days of the cycle prior to surgery. After collection, the blood samples were immediately centrifuged; the serum was removed, placed in a cryotube and frozen at -80 °C. Serum CA125 levels were measured in accordance with the manufacturer’s instructions (BYK-Sangtec Diagnostica GmbH, Germany). When the CA125 values were higher than 35 U/ml, a second measurement was performed to confirm the result.

### Statistical analysis

The Mann-Whitney test was performed to compare the serum CA125 levels among the patients. The significance level was set at 5% (a £ 0.05).

## RESULTS

The histopathological evaluation revealed that 13 (16.25%) out of these 80 patients (without any symptoms of endometriosis like infertility, dysmenorrhea and/or pelvic pain) showed level I or II endometriotic lesions. Of these patients, six (7.5%) had typical and four (5.0%) had atypical lesions. In three patients (3.8%), lesions were found only in the histopathological evaluation study (unapparent endometriosis). In six of these cases, the endometriosis was located in sacrouterine ligaments, and in five in ovarian fossae. In two cases, we found lesions at more than one site. No ovarian endometriosis was observed.

No statistical difference in serum CA125 levels was observed between patients with and without endometriosis: means of 26.9 and 28.3 U/ml, respectively (P = 0.6389 and sample power of 67%).

## DISCUSSION

Endometriosis, a chronic painful inflammatory disease that is one of the most common gynecological disorders,^7^ is defined as the presence of a steroid hormone-dependent endometrium-like tissue consisting of glands and stroma that grows outside the uterine cavity. The target tissues and organs include the fallopian tubes, ovaries, peritoneum, colon, rectovaginal region and bladder.^16^ Endometriosis causes infertility, pelvic pain and dysmenorrhea.^1^^,^^17^ However, up to 20% of women may have endometriosis without presenting any symptoms.^7^ It is a polygenic/multifactorial disease that includes not only hormonal and immunological factors, but also genetic factors.^17^

Considerable efforts have been made towards searching for noninvasive diagnostic methods to detect endometriosis. Moreover, the use of biomarkers has been widely discussed. Abrão et al.^8^ evaluated serum CA125, C-reactive protein, amyloid A protein and anticardiolipin antibodies during the menstrual phase and the middle follicular phase. They found that CA125 was the marker presenting the highest levels during the menstrual phase, between the first and third days of the cycle.

The high levels of CA125 in the bloodstream observed in the presence of an endometriotic ovarian cyst and/or endometriosis with deep infiltration suggest that this antigen may pass into the circulation from endometrial cells in patients with endometriosis.^18^^,^^19^ The CA125 released by the endometrium may reach the blood stream and lymphatic circulation via the peritoneal route, starting from retrograde menstruation, thereby allowing contact with local inflammatory reactions and thus releasing coelomic CA125.^20^ Another explanation for the increased levels of CA125 in the bloodstream could be its access into the abdominal cavity through tubal reflux, thus resulting in absorption by peritoneal lymphatic vessels. Despite the mechanisms proposed, doubts still persist about the real mechanism of CA125 release into the circulation, considering that retrograde menstruation is still controversial and that the levels of this marker change during the postmenopausal period.^20^ Koninckx and Martin^21^ evaluated CA125 and concluded that superficial endometriosis causes elevation of CA125 levels in peritoneal fluid, whereas the deep disease causes their elevation in blood.

Several hypotheses have been raised to explain the cause of elevated serum CA125 at the time of menstruation in patients with endometriosis. They include higher membrane CA125 concentration in ectopic cells than in eutopic endometrial cells;^22^ bleeding relating to eutopic endometrium;^23^ increased transition of CA125 from endometrial tissue to peritoneum, relating to retrograde menses and eutopic endometrium;^22^^,^^23^ an enlarged surface area of endometrial tissue;^24^ inflammatory reaction due to the presence of endometriotic foci; and blood and endometrial shedding into the peritoneal cavity.^23^^,^^24^

Many studies have reported that serum CA125 levels were higher in patients with endometriosis, especially in those at an advanced level, suggesting that monitoring of CA125 in peripheral blood might reflect its behavior in the abdominal environment.^3^^,^^25^

On the other hand, some authors consider superficial endometriosis to be a physiological and intermittent condition in women during their reproductive years, whereas its progression, characterized as deep infiltrative endometriosis and/or endometrial ovarian cysts, is considered to be the true disease.^9^^,^^13^^,^^19^^,^^26^ Divergences persist regarding the natural history of endometriosis, its symptoms, extent, location and staging. The presence of pelvic pain, especially dysmenorrhea, plus infertility and dyspareunia, are the trio that characterizes the disease.^9^

In the present study, 16.25% of the patients had mild endometriosis, and the serum CA125 levels showed no difference between fertile asymptomatic women with and without endometriosis, although the number of cases investigated was small. Moreover, endometriosis was observed in healthy peritoneum of fertile women,^27^ in the same way as reported by Nezhat et al.,^28^ Nisolle et al.^29^ and Balasch et al.^30^ among women with infertility and pelvic pain.

## CONCLUSION

In conclusion, the data suggest that incidental findings of minimal/mild endometriosis may not be of clinical significance, and progression of the disease probably occurs as a result of immunological and genetic abnormalities.The serum CA125 levels in our study did not show any diagnostic significance for detecting the disease.
